# Optimization of Plating Parameters and Properties of Ultrasonic-Assisted Jet-Electrodeposited Ni-W-Al_2_O_3_ Nanocomposite Coatings

**DOI:** 10.3390/ijms26062404

**Published:** 2025-03-07

**Authors:** Mengyu Cao, Dehao Tian, Xue Guo, Wei Li

**Affiliations:** 1 College of Mechanical Science and Engineering, Northeast Petroleum University, Daqing 163318, China; caomyu@126.com (M.C.); 892283668@126.com (D.T.); 2 College of Petroleum Engineering, Northeast Petroleum University, Daqing 163318, China

**Keywords:** Ni-W-Al_2_O_3_ nanocomposite coating, optimization, JMP software, microstructure, wear resistance

## Abstract

Ni-W-Al_2_O_3_ nanocomposite coatings were fabricated using ultrasonic-assisted jet electrodeposition (UAJED) to improve the wear resistance of agricultural machinery parts. To find the best combination of process parameters, the response surface plotter, contour plotter, and pre-set plotter in the JMP (version Pro 14.3.0) software were employed to investigate the effects of various process parameters (jet rate, Al_2_O_3_ content, and ultrasonic power) on the microhardness of the nanocomposite coatings. The surface morphology, microstructure, and properties of the coatings, which were prepared under various combinations of process parameters, were studied through scanning electron microscopy (SEM), an X-ray diffractometer (XRD), transmission electron microscopy (TEM), a microhardness tester, and tribemates to determine the optimal process parameters for creating Ni-W-Al_2_O_3_ nanocomposite coatings. The results indicated that the jet rate, Al_2_O_3_ content, ultrasonic power, interaction terms, and quadratic terms significantly influenced the microhardness of the coatings. The optimized process parameters using the JMP software were a jet rate of 3.71 m/s, Al_2_O_3_ content of 15.38 g/L, and ultrasonic power of 210 W. Furthermore, the coatings produced under these optimal conditions showed low wear rates and friction coefficients, a refined grain size, a dense surface topology, and a high microhardness (724.9 HV).

## 1. Introduction

The production of agricultural products depends heavily on agricultural machinery, but the wear and tear of these parts is becoming more prominent. Parts of agricultural machinery are prone to wear and corrosion during high-intensity agrarian operations, reducing productivity and harming the equipment [1,2,3]. However, the surfaces of agricultural machinery parts can be protected by coating them with a composite coating. Methods for composite coating preparation mainly include chemical deposition (CD), electrodeposition (ED), vapor deposition (VD), and ultrasonic-assisted jet electrodeposition (UAJED). UAJED has many advantages, such as selectivity, low costs, a high deposition efficiency, and a good plating quality. UAJED is frequently used to modify the surfaces of metal components because of these advantages [4,5]. Furthermore, introducing automation and intelligent technology to further improve production efficiency may be another key focus for its future development.

Ni-W alloys with a good stability, high wear resistance, good corrosion resistance, and high tensile strength are widely used in machinery, aerospace, ships, and other fields [6,7,8]. Ni-W alloys are no longer enough to satisfy the demand for materials in a variety of fields due to their rapid development. Some researchers have added nanoparticles with special properties (such as Al_2_O_3_, SiC, and ZrO_2_) to Ni-W alloys [9,10,11]. Al_2_O_3_ nanoparticles are widely employed in the ceramic materials, coating, and catalyst industries due to their high hardness, excellent wear resistance, and good heat resistance. Wang et al. [12] synthesized a Ni-Al_2_O_3_ composite coating via ED on the surfaces of connecting rod axial tiles and found that the coating greatly increased the microhardness and wear resistance of the connecting rod axial tiles. When preparing Ni-W-based coatings through traditional ED methods, such as jet electrodeposition (JED) and pulse electrodeposition (PED), issues such as a poor surface quality and low deposition efficiency frequently emerge. Thanks to the cavitation effect and stirring effect of ultrasound, particles are more evenly distributed in the deposition layer and the deposition rate is accelerated, resulting in a better surface quality of coatings prepared by UAJED.

Process parameters have a great influence on the surface morphology, structure, and properties of composite coatings. The selection of the best process parameters mainly depends on the one-factor test method and the orthogonal test method. Lin et al. [13] investigated the corrosion morphology of a silicon carbide coating using the one-factor test method and determined the process parameters with which the coating performed best. Moreover, An et al. [14] used an orthogonal test method to determine the best process parameters for the preparation of a Ni-W alloy coating. However, such test methods still have limits in forecasting the ideal performance parameters, since the process parameters obtained by these approaches do not always match the optimal process parameters determined by experiments. Compared with these test methods, the least squares method is often used for optimizing experimental parameters due to its simplicity and efficiency in calculation, high accuracy, and wide adaptability. Moreover, the JMP data analysis software is widely used owing to its strong data throughput capability, ease of operation, and diverse functionality. In this research work, Ni-W-Al_2_O_3_ composite coatings were prepared on the surface of Q235 steel via the UAJED method, and the optimal process parameters were determined using the least squares method through the JMP software. The surface shape, structure, and characteristics of the plated layers obtained with various parameters were compared to confirm the accuracy of the optimization results.

## 2. Results and Discussion

### 2.1. Deposition Principle Analysis

**Figure 1:**
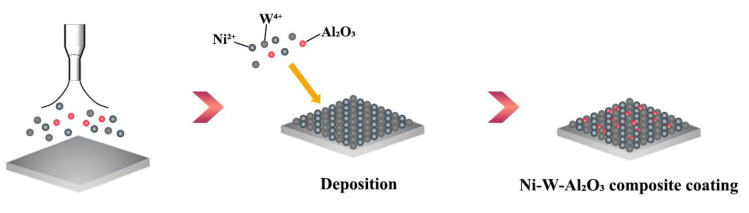
The deposition principle of Ni-W-Al_2_O_3_ composite coatings obtained by UAJED.

Figure 1 shows the deposition principle for the Ni-W-Al_2_O_3_ composite coatings obtained by UAJED. The deposition process for fabricating the Ni-W-Al_2_O_3_ composite coatings is listed as follows: (a) The plating solution was sprayed on the Q235 steel surface. (b) The Ni^2+^ ions, W^4+^ ions, and Al_2_O_3_ nanoparticles in the plating solution were adsorbed on the cathode surface, and the electrochemical reactions are expressed by using Equations (1) and (2). (c) The Ni^2+^ and W^4+^ ions obtained the electrons, and then the composite coating was formed.

(1) $${\mathrm{Ni}}^{2+}+2e\;\text{→}\;\mathrm{Ni}$$

(2) $$W^{4+}+4e\;\text{→}\;W$$

### 2.2. Analysis of Test Results

UAJED was used to prepare the Ni-W-Al_2_O_3_ composite coatings, and the jet rate, Al_2_O_3_ content, and ultrasonic power plating parameters were varied to prepare different coatings. The experimental results of the Ni-W-Al_2_O_3_ composite coatings are illustrated in Table 1. The obtained data were fitted using the least squares method. The coating with the maximum microhardness was developed using the eleventh process parameters, which were adjusted to a G1 combination.

**Figure 2:**
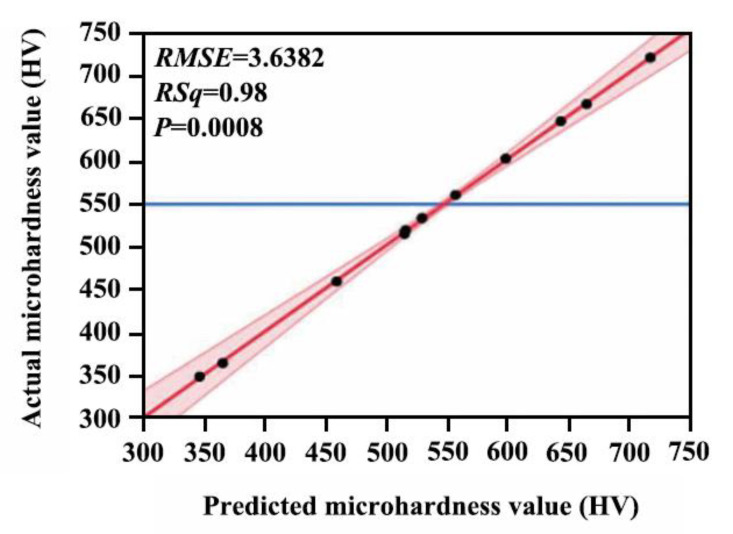
Predicted and actual microhardness values of Ni-W-Al_2_O_3_ composite coatings.

Figure 2 shows the predicted and actual microhardness of the Ni-W-Al_2_O_3_ composite coatings following the least squares fitting method. The actual microhardness is depicted by black dots, the possible area of the predicted value by the red area, and the predicted microhardness by the red solid line with a slope of one. Most of the black dots fall into the red area, indicating that the prediction model is relatively reliable. The blue solid line represents the average value of the microhardness. Furthermore, the *RSq* of the fitted model is 0.98, the *P* is 0.0008, and the *RMSE* is 3.6382, further suggesting that the prediction model can predict the microhardness of composite coatings.

The mathematical model analysis of variance is displayed in Table 2. The linear term, the secondary interaction term, and the secondary power term all showed significant effects on the microhardness. The sum of corrections *p* < 0.05 indicates that the model can predict the microhardness of composite coatings.

### 2.3. Response Surface and Contour Analysis

**Figure 3:**
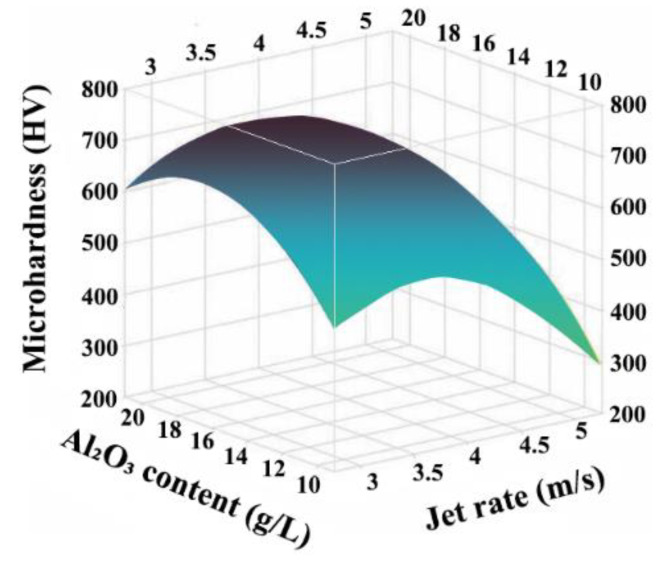
Influence of the interaction between jet rate and Al_2_O_3_ content on the microhardness of Ni-W-Al_2_O_3_ composite coatings.

The relationship between the jet rate, the Al_2_O_3_ content, and the microhardness is illustrated in Figure 3. As the jet rate and Al_2_O_3_ content increased, the microhardness value first increased and subsequently decreased. The microhardness of the Ni-W-Al_2_O_3_ composite coatings reached its highest when the jet rate and Al_2_O_3_ content were 3.6 m/s and 16.7 g/L, respectively. These findings demonstrate that the amount of composite in the coating enhanced as the Al_2_O_3_ content increased. This can improve the microhardness of the composite coating and strengthen the effect of fine crystal reinforcement. However, when the Al_2_O_3_ nanoparticle content was excessively high, the nanoparticles aggregated in the plating solution, reducing the microhardness of the coating, the deposition efficiency, and the composite amount of nanoparticles. Similarly, the efficiency of liquid-phase mass transfer increased with an increase in the jet rate. This could improve the composite amount of nanoparticles, improve the microhardness of the coating, and enable the co-deposition efficiency of nanoparticles and metal cations. Furthermore, the nanoparticles that had not been tightly bonded on the surface were washed into a plating solution as the jet rate increased further, lowering the microhardness of the coating and the composite amount of nanoparticles.

**Figure 4:**
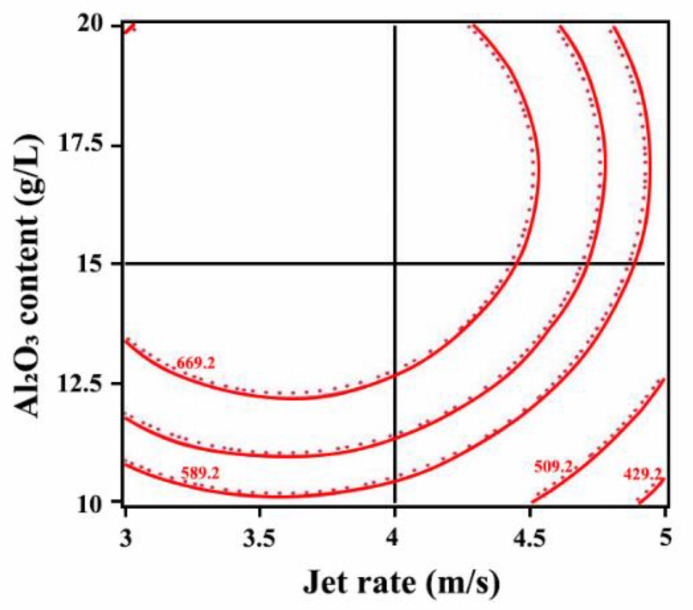
Influence of the interaction of jet rate and Al_2_O_3_ content on the microhardness of Ni-W-Al_2_O_3_ composite coatings.

As shown in Figure 4, the contour lines were elliptical, indicating an interaction between the Al_2_O_3_ content and jet rate. The contour lines were denser along the Al_2_O_3_ content direction, suggesting that the Al_2_O_3_ content dominated the interaction.

**Figure 5:**
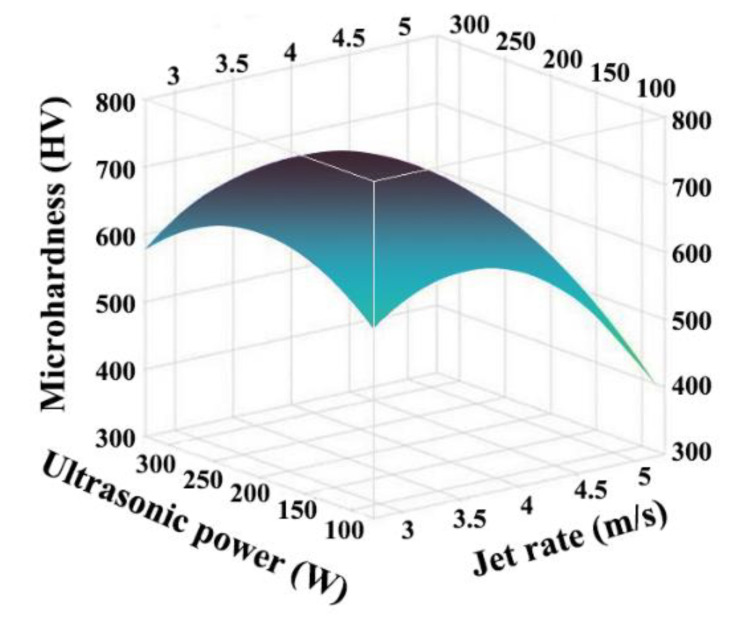
Influence of jet rate and ultrasonic power interaction on microhardness of Ni-W-Al_2_O_3_ composite coatings.

Figure 5 illustrates the relationship between the microhardness, ultrasonic power, and jet rate. The microhardness of the composite coatings reached the maximum when the jet rate and the ultrasonic power were 3.6 m/s, and 210 W, respectively. The results demonstrate that, as the ultrasonic power increased, the ultrasonic shocking effect on the plating solution improved, resulting in a more uniform distribution of nanoparticles, a faster rate of co-deposition, more nanoparticles in the coating, an enhanced effect of fine crystal reinforcement, and a considerable improvement in the microhardness of the composite coatings. As the ultrasonic power increased further, the vibration caused by the ultrasonic wave became too strong, causing the loosely bound nanoparticles to fall off the surface. This decreased the composite amount of nanoparticles and the microhardness of the Ni-W-Al_2_O_3_ composite coatings.

**Figure 6:**
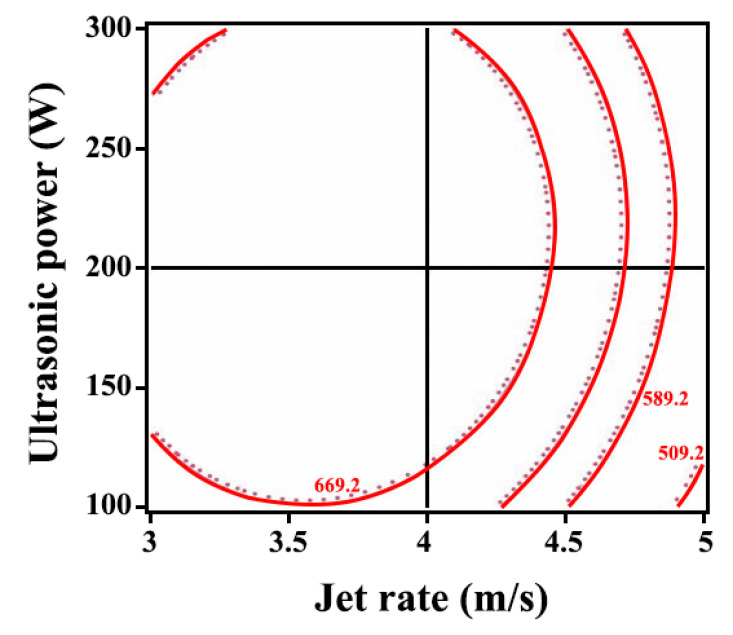
Influence of the interaction of jet rate and ultrasonic power on the microhardness of Ni-W-Al_2_O_3_ composite coatings.

Moreover, the contour lines were elliptical was illustrated in Figure 6, indicating an interaction between the ultrasonic power and jet rate. Furthermore, the contour lines were denser in the jet rate direction, suggesting that the jet rate showed a significant impact on the relationship between the jet rate and ultrasonic power.

**Figure 7:**
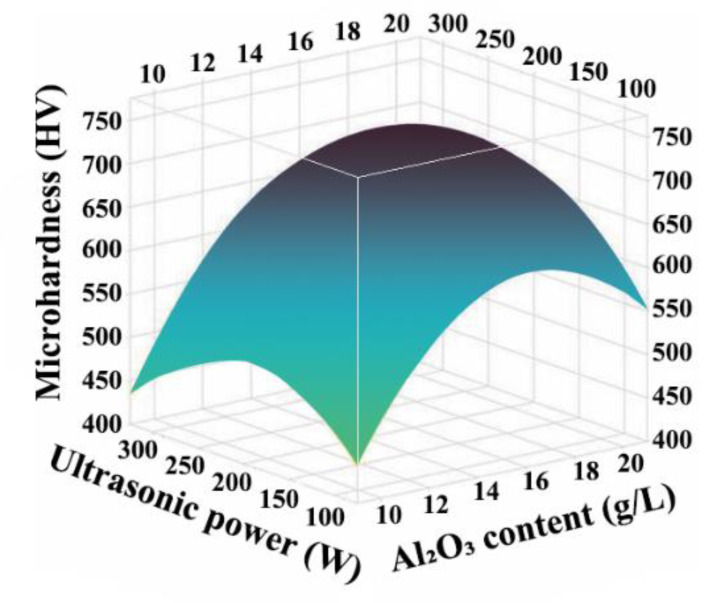
Influence of ultrasonic power and Al_2_O_3_ content on the microhardness of Ni-W-Al_2_O_3_ composite coatings.

**Figure 8:**
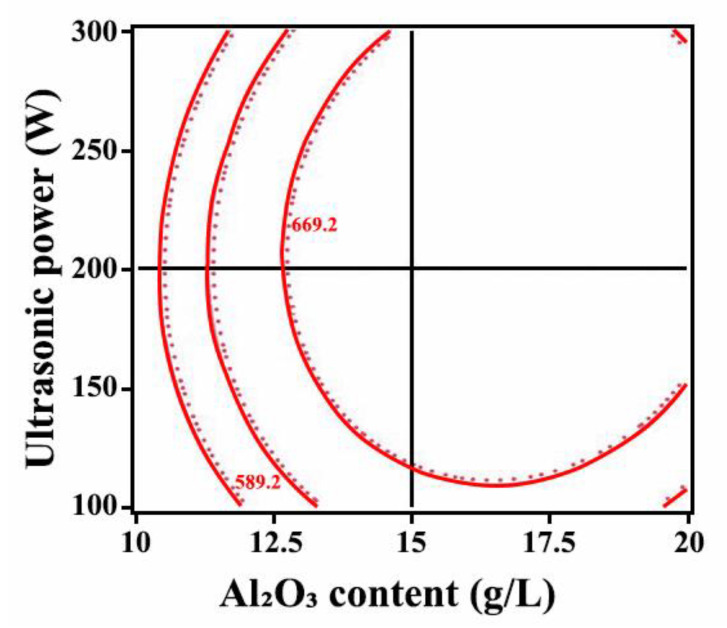
Influence of the interaction of ultrasonic power and Al_2_O_3_ content on the microhardness of Ni-W-Al_2_O_3_ composite coatings.

Figure 7 shows the correlation between the microhardness, ultrasonic power, and Al_2_O_3_ content. The microhardness of the Ni-W-Al_2_O_3_ composite coatings reached the maximum value when the ultrasonic power was 210 W and the content of Al_2_O_3_ nanoparticles was 16.7 g/L. From Figure 8, the contour lines were elliptical, indicating a strong interaction between the Al_2_O_3_ content and ultrasonic power. Moreover, the contours were denser in the direction of Al_2_O_3_ content changes, suggesting that the interaction was affected by the content of Al_2_O_3_ nanoparticles.

**Figure 9:**
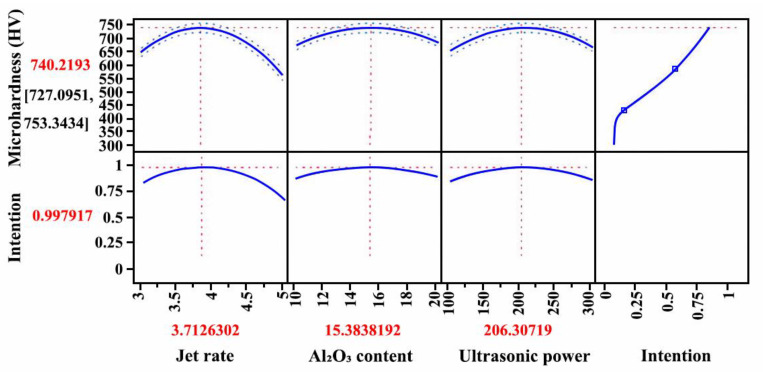
Prediction of maximum microhardness and technological parameters of Ni-W-Al_2_O_3_ composite coatings.

The prediction of the maximum microhardness and technological parameters of the Ni-W-Al_2_O_3_ composite coatings is illustrated in Figure 9. The maximum microhardness was 740.2193 HV predicted by the JMP data analysis software, and the process parameters for the maximum microhardness were a jet rate of 3.71 m/s, an Al_2_O_3_ content of 15.38 g/L, and an ultrasonic power of 206.31 W. The process parameters for the maximum microhardness were set as a G2 combination. It was necessary to adjust the ultrasonic power due to the limitations of the ultrasonic generator, and the corrected power was 210 W. Tests of three groups were carried out to confirm the accuracy of the prediction, and Table 3 shows the microhardness values. The average microhardness value was 725.17 HV, and the value of relative error between the average value and predicted value was 2.1%, suggesting that the model showed the right prediction for the microhardness of the Ni-W-Al_2_O_3_ composite coatings.

### 2.4. Surface Morphology, Organization, and Properties of Ni-W-Al_2_O_3_ Composite Coatings

#### 2.4.1. Surface Morphology Analysis

**Figure 10:**
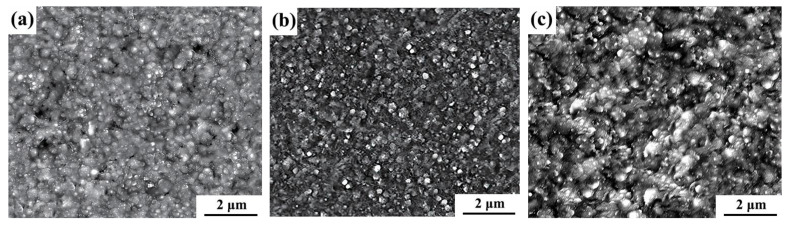
SEM images of Ni-W-Al_2_O_3_ composite coatings prepared via different combinations: (**a**) G1, (**b**) G2, and (**c**) G3.

To verify the accuracy of the optimized process parameters, a new plating parameter combination was set as G3, and the three combinations of plating parameters are displayed in Table 4. Ni-W-Al_2_O_3_ composite coatings were prepared by using three different process parameter combinations of G1, G2, and G3. It was noted that the combination of G1 and G2 was the parameter combination used in actual experiments, while G3 was the parameter combination optimized using the JMP software. The surface morphology of the Ni-W-Al_2_O_3_ coatings was determined through SEM, and Figure 10 displays the corresponding results. The composite coatings fabricated using the G1 and G3 combinations demonstrated an uneven surface with large grain sizes and agglomerations of particles. On the other hand, the G2 combination produced a composite coating with a smooth, flat surface with minute grains, nanoparticles scattered throughout, and a dense structure. It was observed that the limiting current density and co-deposition efficiency of nanoparticles and mental cations were improved by using appropriate plating parameters. More nucleation sites were available for grain development due to the increased composite quantity of Al_2_O_3_ nanoparticles in the coating. Furthermore, the use of suitable plating conditions reduced the hydrogen reaction effect on the surface of the composite coating, increasing the density and homogeneity of the coating [15,16,17,18].

#### 2.4.2. XRD Analysis

**Figure 11:**
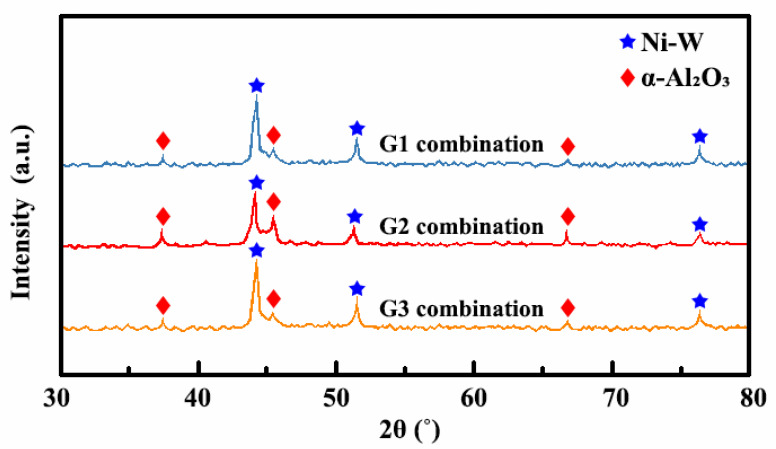
XRD spectrums of Ni-W-Al_2_O_3_ composite coatings.

Figure 11 displays the physical-phase composite of the Ni-W-Al_2_O_3_ composite coatings. All three combinations of plating parameters produced a composite coating with Ni-W and Al_2_O_3_ phases. The main structure of the Al_2_O_3_ phase was α-Al_2_O_3_ and the crystal growth directions of the α-Al_2_O_3_ were (111), (200), and (220), indicating that changes in the plating parameters did not affect the structure and crystal growth directions of the Ni-W-Al_2_O_3_ composite coatings. According to the Scherrer formula, which states that grain size and diffraction peak are inversely related, the composite coating produced with the G1 and G3 combinations exhibited narrow and high Ni-W diffraction peaks, suggesting that the coatings possessed large grain sizes. However, the composite coating produced with the G2 combination demonstrated short and broad Ni-W diffraction peaks, indicating a high degree of grain refinement and a small grain size.

(3) $$D=\frac{K\lambda }{\beta cos\theta }$$

where *D* represents the grain size (nm), *K* denotes Scherrer’s constant (*K* = 0.89), *λ* = X-ray wavelength (*λ* = 1.54056 Å), *β* = the half-height width of the diffraction peak of the measured sample(rad), and *θ* = the diffraction angle of the corresponding diffraction peak (°).

The average particle sizes of the Ni-W solution and the Al_2_O_3_ nanoparticles were determined through Equation (3), and the average particle sizes of the composite coatings prepared by different plating parameter combinations are displayed in Table 5. The phase content is represented by the area of the diffraction peaks. The composite coating produced via the G1 and G3 combinations displayed short Al_2_O_3_ diffraction peaks, suggesting that the Ni-W grain size was large and the composite amount of Al_2_O_3_ nanoparticles was low. Nevertheless, the intense Al_2_O_3_ diffraction peaks suggested that the coating contained a significant composite quantity of Al_2_O_3_ nanoparticles.

#### 2.4.3. TEM Analysis

**Figure 12:**
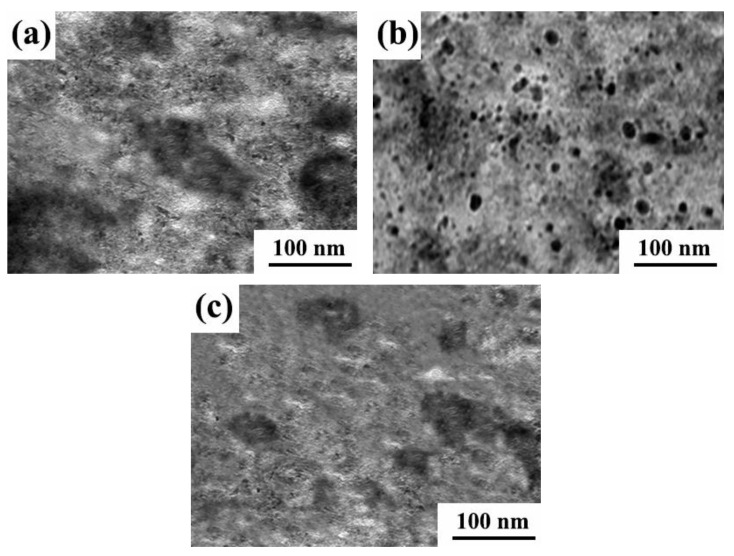
TEM images of Ni-W-Al_2_O_3_ composite coatings prepared with different parameter combinations: (**a**) G1, (**b**) G2, and (**c**) G3.

Figure 12 shows the TEM of the Ni-W-Al_2_O_3_ composite coatings prepared using various parameter combinations. The nanoparticle agglomeration phenomenon was caused by the composite coating produced through the G1 and G3 parameter combinations. Furthermore, the Al_2_O_3_ nanoparticles were distributed in blocks and the diameter of the Al_2_O_3_ nanoparticles was large. However, the nanoparticles of the coating prepared through the G2 parameter combination were uniformly dispersed with a smaller nanoparticle size. It was observed that employing suitable plating parameters successfully prevented nanoparticle agglomeration, enhanced the composite amount of nanoparticles, and improved the co-deposition of nanoparticles and metal cations. Finally, as the amount of nanoparticle composites in the coating increased, the small size effect of the nanoparticles was enhanced, the excessive growth of the grain was inhibited, and the grain size was reduced.

**Figure 13:**
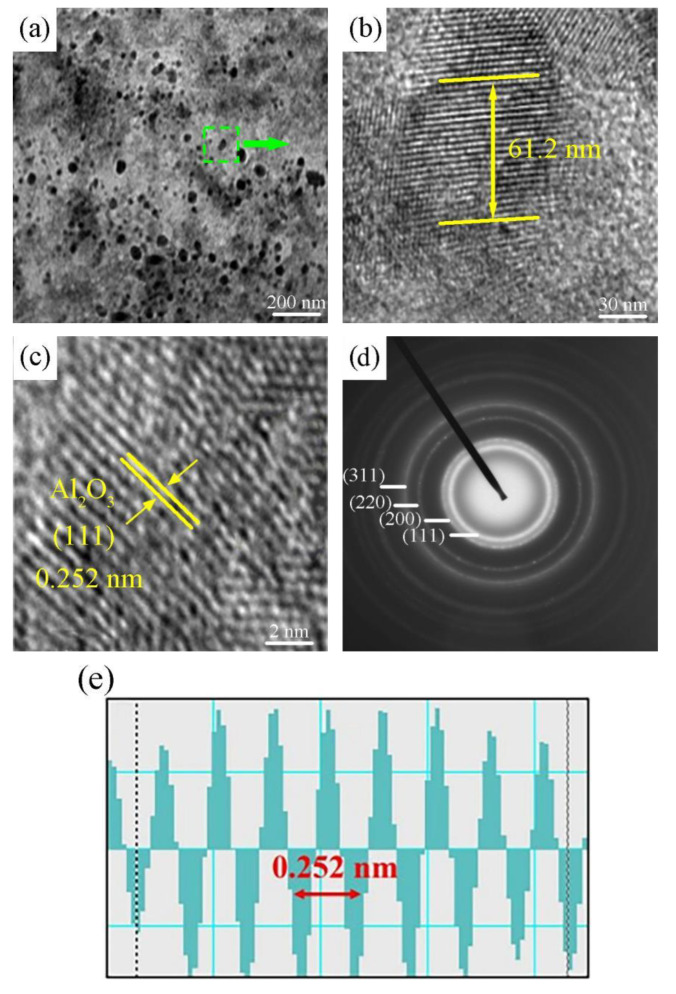
The obtained TEM images for the Ni-W-Al_2_O_3_ coatings prepared with G2 process parameters combination: (**a**) low magnification, (**b**) higher magnification, (**c**) lattice fringes of Al_2_O_3_ nanoparticles, (**d**) selected area electron diffraction (SAED) pattern, and (**e**) noise-filtered image of Al_2_O_3_ nanoparticles.

The Ni-W-Al_2_O_3_ composite coating prepared through the G2 process parameter combination displayed an improved performance. Therefore, TEM was used to further study the Ni-W-Al_2_O_3_ composite coating fabricated using the combination of G2 process parameters; the results are displayed in Figure 13. The TEM image in Figure 13a provides an overview of the composite structure at the nanoscale. The Ni-W grains showed a uniform, thick, and well-organized appearance. The selected area (indicated by the green dashed box) highlights the distribution of Al_2_O_3_ nanoparticles within the Ni-W matrix, suggesting the uniform incorporation of Al_2_O_3_ into the Ni-W composite. In Figure 13b, a higher-magnification TEM image of the selected region reveals the finer microstructural details of the Ni-W-Al_2_O_3_ composite coating. Al_2_O_3_ nanoparticles with a measured size of 45.3 nm were observed within the Ni-W matrix. The particles displayed distinct contrast, indicating the presence of crystalline phases. The microstructure appeared dense, with no obvious voids or defects.

The TEM image in Figure 13c further elucidates the crystalline nature of the Al_2_O_3_ nanoparticles. The lattice fringes, with a measured interplanar spacing of 0.252 nm, correspond to the (111) plane of Al_2_O_3_. The clear lattice fringes indicate that the Al_2_O_3_ nanoparticles retained their crystallinity during the electrodeposition process, with minimal distortion. The diffraction rings (SAED pattern, Figure 13d) correspond to the (111), (220), and (311) planes of Al_2_O_3_, indicating the presence of crystalline Al_2_O_3_ within the Ni-W matrix. Moreover, Figure 13e displays the corresponding lattice spacing data, which correlate with the TEM findings. The periodic lattice fringes and the interplanar spacing of 0.252 nm further confirm the presence of the Al_2_O_3_(111) crystal plane. This finding also correlates with the findings of SAED.

#### 2.4.4. Adhesion Performance and Microhardness Analysis

**Figure 14:**
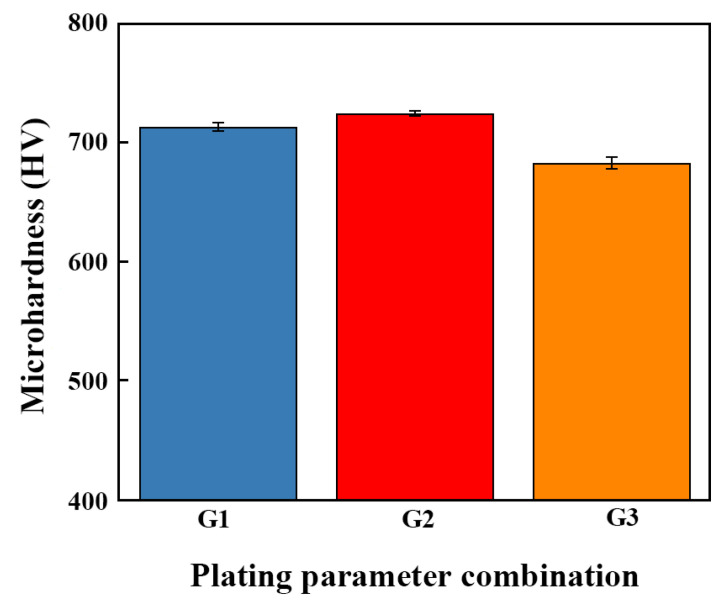
Microhardness values of Ni-W-Al_2_O_3_ composite coatings prepared with different plating combinations.

The plated parts were boiled in boiling water for 0.5 h, then submerged in ice water at 0–5 °C for 5 min, and subsequently removed. After undergoing this cycle five times, the parts were dried. The absence of peeling, cracking, or flaking off of the coatings indicated that all coatings were well-bonded to the substrate and exhibited a strong adhesion performance. A microhardness tester was used to determine the microhardness of the composite coatings, and the results are shown in Figure 14. The microhardness of the Ni-W-Al_2_O_3_ composite coatings fabricated through the G1, G2, and G3 parameter combinations was found to be 713.7, 724.9, and 683.4 HV, respectively. It was found that employing the proper plating parameters greatly increased the co-deposition efficiency and composite amount of the coating.

Al_2_O_3_ nanoparticles can serve as heterogeneous nucleation cores, promoting the recrystallization process of plated metal, further refining the grain size and consequently enhancing microhardness of a coating. During the deposition process, interface reactions occurred between Al_2_O_3_ and the Ni-W matrix, leading to the formation of interface compounds or solid solutions, which enhanced the bonding force between particles and the matrix, thereby improving the cohesive properties and hardness of the coatings. Moreover, increases in the amount of nanoparticle composites resulted in improvements in the microhardness of the composite layer, the nucleation point of the grains, the nucleation rate, and the fine crystal reinforcement [19].

#### 2.4.5. Wear Resistance Analysis

**Figure 15:**
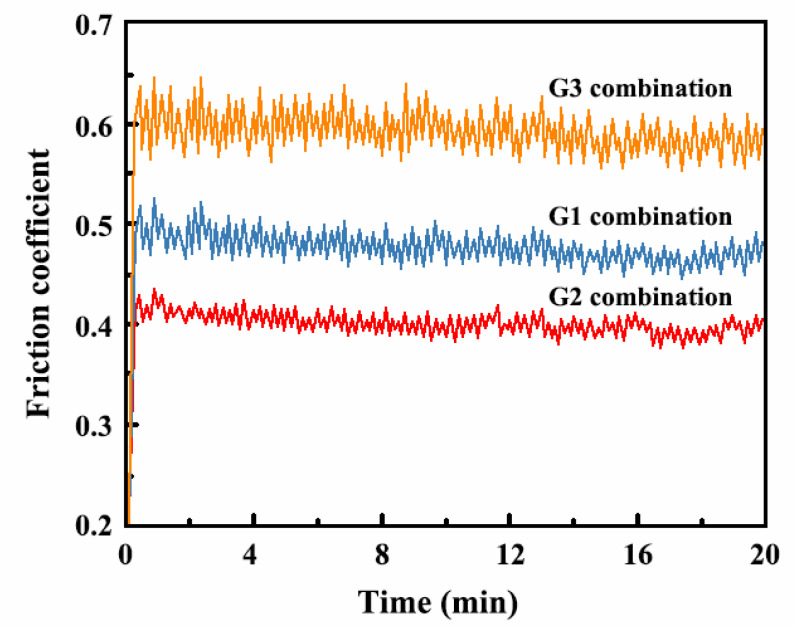
Wear coefficients of Ni-W-Al_2_O_3_ composite coatings prepared with different parameter combinations.

A friction wear tester was used to measure the friction coefficient, and Figure 15 displays the results. The friction coefficients of G1 and G3 were found to be 0.54 and 0.62, respectively. The fluctuation in the friction coefficient curves of G1 and G3 was strong, indicating that the wear resistance of the coating was poor and the surface of the coating was uneven. However, the friction coefficient curve of G2 was relatively smooth and the friction coefficient of G2 was found to be 0.41, suggesting that the wear resistance of the coating was improved.

**Figure 16:**
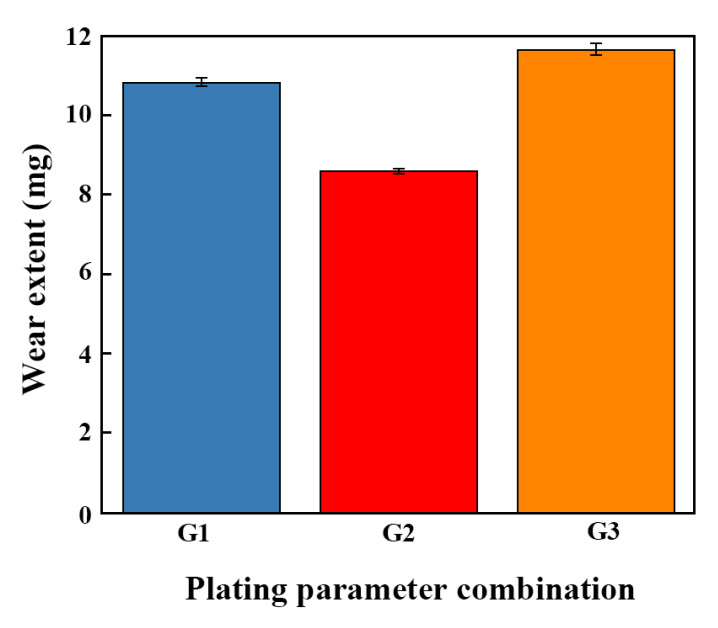
Wear extents of Ni-W-Al_2_O_3_ composite coatings prepared with different parameter combinations.

Figure 16 depicts the wear contents of the Ni-W-Al_2_O_3_ composite coatings deposited using different parameter combinations. The wear amounts of G1, G2, and G3 were found to be 10.84, 8.61, and 9.67 mg, respectively. The great hardness and strength of Al_2_O_3_ nanoparticles as a reinforcing phase are advantageous for enhancing the properties of coatings. Al_2_O_3_ nanoparticles can serve as solid lubricants, filling the minute gaps between friction interfaces during the tribological process to reduce friction and wear. These particles can also alter the contact state of the friction interfaces, thereby reducing the coefficient of friction. Meanwhile, the tight bonding between Al_2_O_3_ particles and the Ni-W matrix, coupled with strong cohesive properties, also contributed to reducing wear and peeling phenomena. In this study, the Al_2_O_3_ nanoparticle composite amount of G2 was greater than the amounts of G1 and G2, suggesting that the coating produced using the G2 parameter combination showed lower wear and friction coefficients.

## 3. Materials and Methods

### 3.1. Testing of Device and Cathode Materials

**Figure 17:**
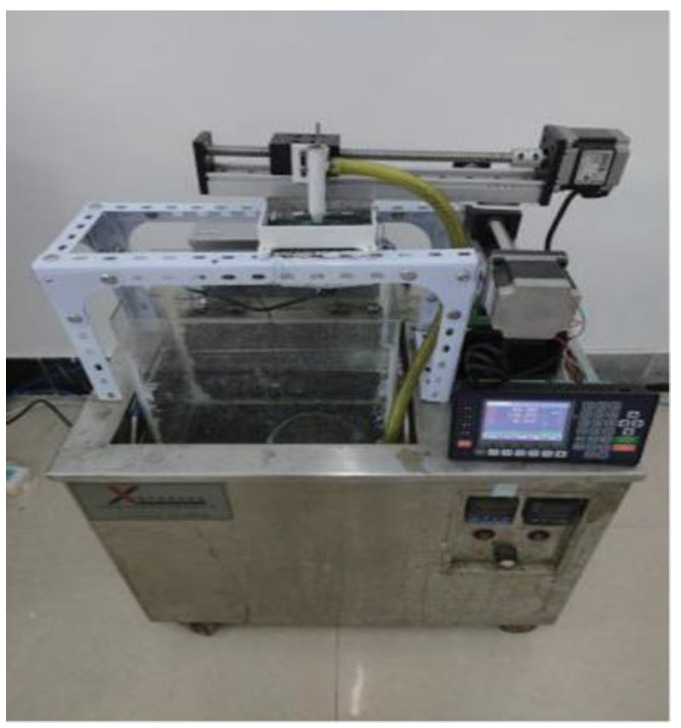
Physical device for Ni-W-Al_2_O_3_ composite coatings fabricated by UAJED.

Figure 17 depicts the UAJED device, which primarily comprised a digital display controller, circulating pumps, an extrusion nozzle, a flowmeter, a cathode specimen, a ball screw, a stepping motor, and an ultrasonic generator. A pure nickel bar with a size of Φ4 mm × 50 mm was used as the anode and the Q235 steel sheet was used as the cathode. The formulation of the plating solution of the Ni-W-Al_2_O_3_ composite coating is elaborated in Table 6. Al_2_O_3_ nanoparticles and sodium dodecyl sulfate were combined in a beaker and mixed with 70 °C distilled water. Thereafter, the mixture was introduced into the plating solution. An MYP11-2A constant-temperature magnetic stirrer (Shanghai Meiyingpu Instrument And Meter Manufacturing Co., Ltd, Shanghai, China) was used to agitate the plating solution before testing.

### 3.2. Characterization

The surface morphology of the Ni-W-Al_2_O_3_ composite coatings was observed through an FSEM-S4800 scanning electron microscope (SEM, Hitachi Limited, Tokyo, Japan). The organizational structure and grain growth direction of the Ni-W-Al_2_O_3_ composite coatings were studied using an X-ray diffractometer (XRD, D/max-2000, Science and Technology Co., Ltd, Tokyo, Japan). The microhardness of the Ni-W-Al_2_O_3_ composite coatings was explored using an HV-1MD automatic turret digital microhardness tester (Shanghai Biaoyu Precision Instrument Co., Ltd, Shanghai, China), and five randomly selected test points were used to obtain the microhardness average. The friction coefficient of the Ni-W-Al_2_O_3_ composite coatings was measured using a CETR-3 friction and wear tester (Bruker (Beijing) Technology Co., Ltd, Beijing, China). An XPR105DR/AC electronic analytical balance (Mettler Toledo (China) Technology Co., Ltd, Changzhou, China) was used to measure the wear content of the samples. Moreover, a JEM-1400Plus transmission electron microscope (TEM, JEOL Japan Electronics Co., Ltd, Tokyo, Japan) was used for examining the microstructure of the Ni-W-Al_2_O_3_ composite coatings.

### 3.3. Experimental Design

In this study, the jet rate (*x*_1_), Al_2_O_3_ content (*x*_2_), and ultrasonic power (*x*_3_) of UAJED-fabricated Ni-W-Al_2_O_3_ composite coatings were selected as factors, and microhardness (Y) was selected as a response. The response Y was set as the maximization target. For each factor, three values were selected as its levels, as listed in Table 7. The level was the value of the microhardness of the coating. The coded values of the factors were *X*_1_ = (*x*_1_ − 4)/1, *X*_2_ = (*x*_2_ − 15)/5, *X*_3_ = (*x*_3_ − 200)/100.

## 4. Conclusions

Changes in jet rate, Al_2_O_3_ content, and ultrasonic power demonstrated significant effects on the microhardness of Ni-W-Al_2_O_3_ composite coatings. The optimized plating parameters using the JMP software were a jet rate of 3.71 m/s, an Al_2_O_3_ content of 15.38 g/L, and an ultrasonic power of 210 W. The Ni-W-Al_2_O_3_ composite coating fabricated through the optimal plating parameters revealed an outstanding wear resistance and improved microhardness.

## Figures and Tables

**Table 1:** Experimental results for producing Ni-W-Al_2_O_3_ composite coatings.

Sample Number	*X*_1_	*X*_2_	*X*_3_	*Y*
1	0	−1	1	517.9
2	−1	1	−1	600.4
3	1	1	1	531.9
4	0	1	1	663.3
5	1	−1	1	364.8
6	1	−1	−1	349.2
7	−1	−1	−1	513.7
8	1	1	−1	458.8
9	−1	−1	0	558.5
10	−1	1	0	664.3
11	0	0	0	716.8
12	−1	0	1	643.5

**Table 2:** Mathematical model analysis of variance.

Source	Degree of Freedom	Sum of Squares	*F* Ratio	*p* Value
*X*_1_	1	29,386.411	2220.1320	0.0005 *
*X*_2_	1	37,773.316	2853.7600	0.0004 *
*X*_3_	1	907.761	68.5810	0.0143 *
*X*_1_ *X*_2_	1	405.536	30.63810	0.0311 *
*X*_1_ *X*_3_	1	610.090	46.0921	0.021 *
*X*_2_ *X*_3_	1	1207.136	91.1987	0.0108 *
*X*_1_ *X*_1_	1	13,577.926	1025.8070	0.001 *
*X*_2_ *X*_2_	1	8221.158	621.1054	0.0016 *
*X*_3_ *X*_3_	1	5117.293	386.6095	0.0026 *
Model	9	150,640.840	1264.5400	—
Error	2	26.470	—	—
Corrected sum	11	150,667.310	—	0.0008 *

Note: *p* < 0.05 means the item has a significant effect on the response. “*” indicates *p* < 0.05.

**Table 3:** Microhardness values of Ni-W-Al_2_O_3_ composite coatings.

Experiment Number	Microhardness (HV)
1	726.1
2	724.4
3	725.0
Average value	725.17
Predicted value	740.2193

**Table 4:** Plating parameters of G1, G2, and G3 combination.

Combination	Jet Rate (m/s)	Al_2_O_3_ Content (g/L)	Ultrasonic Power (W)
G1	4	15	200
G2	3.71	15.38	210
G3	3	15	250

**Table 5:** Average particle sizes of Ni-W-Al_2_O_3_ composite coatings.

	Ni-W (nm)	Al_2_O_3_ (nm)
G1	67.1	41.6
G2	57.4	24.3
G3	76.9	59.4

**Table 6:** Composition of the plating solution for producing Ni-W-Al_2_O_3_ composite coatings.

Element	Content (g/L)
NiSO_4_·6H_2_O	260
NiCl_2_·6H_2_O	30
Na_2_WO_4_·2H_2_O	25
H_3_BO_4_	30
Na_3_C_6_H_5_O_7_	20
C1_2_H_25_NaSO_4_	0.2
Al_2_O_3_ nanoparticle	10~20

**Table 7:** Factors and levels for producing Ni-W-Al_2_O_3_ composite coatings.

Level	Factors		
	Jet Rate *x*_1_ (m/s)	Al_2_O_3_ Content *x*_2_ (g/L)	Ultrasonic Power *x*_3_ (W)
−1	3	10	100
0	4	15	200
1	5	20	300

## Data Availability

The original contributions presented in this study are included in the article. Further inquiries can be directed to the corresponding author.
